# Manual on the proper use of yttrium-90-labeled anti-P-cadherin antibody injection for radionuclide therapy in clinical trials

**DOI:** 10.1007/s12149-019-01409-x

**Published:** 2019-10-12

**Authors:** Makoto Hosono, Hideharu Ikebuchi, Seigo Kinuya, Sachiko Yanagida, Yoshihide Nakamura, Takahiro Yamada, Kenta Sakaguchi, Hiroyasu Sugano, Kiyotaka Kojima, Jun Hatazawa

**Affiliations:** 1 Department of Radiology, Faculty of Medicine, Kindai University, 377-2 Ohno-Higashi, Osaka-Sayama, Osaka 589-8511 Japan; 2 Japanese Society of Nuclear Medicine, Tokyo, Japan; 3 Japan Radioisotope Association, Tokyo, Japan; 4 Atomic Energy Research Institute, Kindai University, Higashi-Osaka, Japan; 5 FUJIFILM Toyama Chemical Co., Ltd., Tokyo, Japan

**Keywords:** Yttrium-90-labeled anti-P-cadherin antibody, Radionuclide therapy, Radiation protection

## Abstract

We present the guideline for use of yttrium-90-labeled anti-P-cadherin antibody injection for radionuclide therapy in clinical trials on the basis of radiation safety issues in Japan. This guideline was prepared by a study supported by the Ministry of Health, Labour, and Welfare, and approved by the Japanese Society of Nuclear Medicine. Treatment using yttrium-90-labeled anti-P-cadherin antibody injection in Japan should be carried out according to this guideline. Although this guideline is applied in Japan, the issues for radiation protection shown here are considered internationally useful as well. Only the original Japanese version is the formal document.

## Goals of radiation safety management

This manual covers radiation safety management in conjunction with the use of yttrium-90-labeled anti-P-cadherin antibody injection (hereinafter referred to as “this drug”) for P-cadherin-positive solid cancer in a clinical trial (hereinafter referred to as “this clinical trial”), and this manual was compiled to comply with the principles of safety guidelines related to “Release of Patients who have been Administered a Radiopharmaceutical” (Notification No. 70 from the Safety Division, Pharmaceutical and Medical Safety Bureau, the Ministry of Health, Labour, and Welfare dated June 30, 1998, hereinafter referred to as “PMSB Notification No. 70”) [1], which was amended by “Release of Patients who have been Administered a Radiopharmaceutical” (Notification No. 0511-1 from the Medical Care Planning Division, Health Policy Bureau dated May 11, 2016, hereinafter referred to as “HPB Notification No. 0511-1”) [2]. This manual was also compiled to ensure the safe handling of this drug.

To safely perform internal radiotherapy with this drug (hereinafter referred to as “this therapy”) in clinical trials, it is essential to ensure the safe handling of radiopharmaceuticals, prevention of radiation exposure, and prevention of contamination. In particular, it is important to pay sufficient attention not only to persons involved such as patients and their families, but also to the general public concerning the safety of radiation.

This manual incorporates the intent of the Medical Care Act [3] and recommendations on radiation protection by international organizations [4–7], and so hospitals or other medical facilities should perform this clinical trial in accordance with the requirements to ensure radiation safety that are covered in this manual.. On the other hand, though a radionuclide used for this drug is yttrium-90 (hereinafter referred to as ^90^Y), a nuclide that emits beta rays as with conventional radioimmunotherapy for non-Hodgkin’s lymphoma, it is assumed that ^90^Y with a high radioactivity level that has not been previously handled in medical institutions will be used for in-hospital labeling of this drug. Therefore, it is important that a hospital or other medical facility conducting this clinical trial becomes familiar with the physical and chemical properties of ^90^Y prior to handling this drug. Also, in the conduct of this clinical trial, in consideration of the fact that a part of ^90^Y is excreted into the urine and the characteristics of beta rays, measurement should be performed with sufficient attention to the handling of this nuclide.

Accordingly, the following points are summarized in this manual on radiation safety management:

1. Guidelines for facility management.
2. Protection from exposure.
3. Storage and disposal of radioactive contaminants from medical sources.

In addition, facilities performing his clinical trial need to meet the following criteria:

1. A hospital or other medical facility conducting this clinical trial will meet criteria for radiological protection in medicine as stipulated in relevant laws and ordinances and follow all legal and regulatory procedures.
2. This clinical trial will be conducted at a hospital or other medical facility employing full-time physicians and radiology technologists with sufficient knowledge of and experience of handling radiopharmaceuticals. In addition, this trial will be conducted at a hospital or other medical facility employing physicians with sufficient knowledge and experience of treating P-cadherin-positive recurrent solid cancer targeted in the trial.
3. This clinical trial will be conducted at a hospital or other medical facility employing a physician who has completed the prescribed education and training specified in this manual.

In Section 5. Precautions (3) in the Appendix of PMSB Notification No. 70, it is described that “Refer to guidelines, etc. prepared by organizations such as radiology societies for protection according to physical properties of radionuclides, explanation to patients and caregivers, and other safety management.”

## Guidelines for facility management

### Characteristics and legal positioning

#### Physical properties of ^90^Y

The physical characteristics of ^90^Y are shown in Table 1.

**Table 1:** Physical properties of ^90^Y (Partial revision of Radioisotope Pocket Data Book [11th edition])

Nuclide	Half-life	Type of decay	Maximum energy (MeV) of β rays and percentage emitted	Photon energy (MeV) and percentage emitted	Effective dose rate constant (μSv m^2^ MBq^−1^ h^−1^)
^90^Y	64.00 h (2.67 days)	β^−^	2.280–100%	None	0.00263^a^

^a^Effective dose rate constant of bremsstrahlung radiation against target of atomic number 20 (From Operating Manual for Calculating the Shield in Radiation Facilities 2015 [27])

^90^Y is disintegrated by β^-^decay with a physical half-life of 64.00 h (2.67 days) emitting only beta rays with the maximum energy of 2.28 MeV. Because beta rays emitted from ^90^Y have high energy, attention should be paid to the bremsstrahlung radiation from the beta rays (see Table 1). It has been shown that the average range in tissues is approximately 2.5 mm (maximum 11 mm) [8].

#### In vivo dynamics of ^90^Y

It has been reported that ^90^Y is hardly taken up from the gastrointestinal tract into the blood after oral intake, and that after intravenous administration, 25% of ^90^Y is directly excreted, 50% is transferred to the skeleton, 15% is transferred to the liver, and 10% is distributed to all other organs and tissues and remains in the body for a long time [9].

#### Exposure dose of this drug

Since ^90^Y is a pure beta emitter, it is not possible to directly evaluate the body distribution of this drug in humans. However, in an interim analysis (*N* = 13) using planar images over time after administration of indium-111-labeled anti-P-cadherin antibody, which was performed in the US phase I clinical trial, the accumulation has been shown to be high in the spleen, kidneys, testes, lungs, and liver in this order among tissues other than tumors, with the exposure doses of 27.0 mGy/MBq, 6.36 mGy/MBq, 5.80 mGy/MBqs, 4.6 mGy/MBqs, and 4.4 mGy/MBq, respectively [10].

#### Relevant laws, ordinances, rules, and regulations

When using a pharmaceutical in treatment as stipulated in Article 2, Section  1 of the Pharmaceuticals and Medical Devices Law, applicable laws, ordinances, rules, and regulations to prevent radiation injuries are generally the following:

1. Medical Care Act [3] (Ordinance for Enforcement of the Medical Care Act [11]): Ministry of Health, Labour, and Welfare.
2. Pharmaceuticals and Medical Devices Act: Ministry of Health, Labour, and Welfare.
3. Medical Practitioners Act: Ministry of Health, Labour, and Welfare.
4. Pharmacists Act: Ministry of Health, Labour, and Welfare.
5. Radiologic Technologists Act: Ministry of Health, Labour, and Welfare.
6. Act on Clinical Laboratory Technicians: Ministry of Health, Labour, and Welfare.
7. Industrial Safety and Health Act (Ordinance on Prevention of Injuries from Ionizing Radiation [12] (hereinafter referred to as the “Ionizing Radiation Ordinance”) and Working Environment Measurement Act): Ministry of Health, Labour, and Welfare.
8. National Public Service Act (Rules of the National Personnel Authority 10-5) [13]: National Personnel Authority.

#### Legal definitions

^90^Y used in this therapy is specified with different terms by law.

In the Medical Care Act, it is classified as a “medical radionuclide” and as “radioactive material” in Rules of the National Personnel Authority 10-5 and Ionizing Radiation Ordinance

1. Article 24, Section 8 Subsection 2 of Ordinance for Enforcement of the Medical Care Act: Medical radionuclides.
2. Rules of the National Personnel Authority 10-5 Article 3, Paragraph 2: Radioactive materials.
3. Article 2, Section 2 of Ionizing Radiation Ordinance: Radioactive materials.

### Requirements for trial site (legal requirements)

#### Requirements for trial site

^90^Y is a “medical radionuclide” specified in the Medical Care Act, the Ordinance for Enforcement of the Medical Care Act, etc.

If a hospital or clinic intendeds to possess a medical radionuclide, the following matters should be notified in advance to the prefectural governor where the hospital or clinic is located in accordance with Article 15, Section 3 of the Medical Care Act and Articles 24 and 28 of the Ordinance for Enforcement of the Medical Care Act:

- Name and location of the hospital or clinic.
- Types, shapes and quantities of medical radionuclides that are scheduled to be used in the year.
- Planned maximum storage quantity, planned maximum quantity used in 1 day, and planned maximum quantity used in 3 months for each type of medical radionuclides.
- Overview of buildings and facilities and precautions for the prevention of radiation injuries of the room where medical radionuclides are used, storage facilities, transportation containers, disposal facilities, and rooms where patients treated with medical radionuclides are admitted.
- Names of physicians using medical radionuclides and their background for radiology.

When a facility where nuclear medicine tests are performed newly adds ^90^Y to medical radionuclides that has been notified to be used, it should be confirmed whether the criteria related to the amount of external radiation and the concentration in the air, exhaust, and drain water are satisfied even after the use of ^90^Y is started, and the change of the nuclides used should be notified in advance.

Also, the requirement related to the prevention of radiation injuries of buildings and facilities are specified in Article 30, Section 8 through Article 30, Section 12 of the Ordinance for Enforcement of the Medical Care Act. In addition, Article 30, Section 13 through Article 30, Section 25 of the Ordinance for Enforcement of the Medical Care Act specifies the obligation for the administrator of a hospital or clinic to comply with in handling medical radionuclides (see Table 2).

**Table 2:** Criteria for dose limits and concentration limits in rooms where medical radionuclides are used

Rooms where medical radionuclides are used	Medical Care Act
Rooms where medical radionuclides are used	Rooms where medical radionuclides are used^a^
	Storage facilities^b^
	Disposal facilities^c^
	Rooms for patients undergoing radiation therapy^d^
Dose limits and concentration limits in controlled areas^e^	The effective dose of external radiation^f^: 1.3 mSv every 3 months Concentration of radioactive isotopes (hereinafter referred to as RIs) in the air^f^: the average concentration over 3 months is 1/10 of the concentration limit of an RI in the air Surface density of a material contaminated with an RI^f^: 1/10 of the surface concentration limit (RIs that do not emit alpha rays: 4 Bq/cm^2^)
Dose limits and concentration limits at places in facilities using RIs where people are constantly entering^a–c^	The effective dose on the external side of walls, etc.: 1 mSv or less every week Concentration of an RI in the air^f^: The average concentration over 1 week is equal to the concentration limit of an RI in the air Surface density of a material contaminated with an RI^f^: Surface concentration limit (RIs that do not emit alpha rays: 40 Bq/cm^2^)
Dose standards at boundaries in a hospital or other medical facility (including areas in the hospital where people stay)^g^	The effective dose is 250 μSv or less^f^ every 3 months
Exposure dose for inpatients^h^	The effective dose does not exceed 1.3 mSv every 3 months

^a^Article 30, Section 8 of the Ordinance for Enforcement of the Medical Care Act: Rooms where medical radionuclides are used

^b^Article 30, Section 9 of the Ordinance for Enforcement of the Medical Care Act: Storage facilities

^c^Article 30, Section 11 of the Ordinance for Enforcement of the Medical Care Act: Disposal facilities

^d^Article 30, Section 12 of the Ordinance for Enforcement of the Medical Care Act: Rooms for patients undergoing radiation therapy

^e^Article 30, Section 16 of the Ordinance for Enforcement of the Medical Care Act: Controlled areas

^f^Article 30, Section 26 of the Ordinance for Enforcement of the Medical Care Act: Concentration limit, etc

^g^Article 30, Section 17 of the Ordinance for Enforcement of the Medical Care Act: Protection at property lines

^h^Article 30, Section 19 of the Ordinance for Enforcement of the Medical Care Act: Prevention of patient exposure

Patients treated with this drug may be released or discharged from the RI-controlled facilities if the requirements of “4.5.1.5 Criteria for release of patients administered this drug from RI-controlled facility, etc.” of this manual are satisfied.

#### Notification regarding the quantity of medical radionuclides to be used

Regarding the “Quantity of medical radionuclides to be used” specified in the Article 28 of the Ordinance for Enforcement of the Medical Care Act, the ordinance stipulates that [2] planned maximum quantity used in 1 day, [1] planned maximum quantity used in 3 months, [3] planned annual quantity used, and [4] planned maximum storage quantity should be notified, and a hospital or other medical facility is not allowed to use them beyond the quantities which have been notified (Article 28 and Article 29, Section 2 of the Ordinance for Enforcement of the Medical Care Act, “Handling of medical radiation at hospitals or clinics” [March 15, 2019, Notification No. 0315-4 from the Health Policy Bureau (HPB), hereinafter referred to as “HPB Notification No. 0315-4”] [14]. The quantity of medical radionuclides to be used must be determined in consideration of the compliance with the standards for buildings and facilities based on the Ordinance for Enforcement of the Medical Care Act. Examples of common methods for determining the quantity to be notified are shown below:

1. Planned maximum quantity used in 1 day: This will be set based on the maximum dose for one patient × maximum number of patients treated in 1 day. Planned maximum quantity used in 1 day can be set by determining the number of tests or treatments per day and per week, taking into account the number of procedures per week or per month based on the dosing regimen.
2. Planned maximum quantity used in 3 months: This will be set based on the maximum planned quantity used in 1 week (maximum number of patients planned in 1 week × maximum dose for 1 patient) × 13 (week/3 months). The 3-month period is specified as the 3 months starting from April 1, July 1, October 1, and January 1, respectively, in the HPB Notification No. 0315-4.
3. Planned annual quantity used: Generally, this is determined as the maximum planned quantity used in 3 months × 4.
4. Planned maximum storage quantity: This is the quantity of several times the planned maximum quantity used in 1 day.

In any case, calculation should be performed in consideration of the packaging unit of the medical radionuclides (for this drug, 1850 MBq/vial i) and the regimen (for this drug, 925/MBq/m^2^/dose [max: 2220 MBq] up to four times per year at intervals of 12 weeks or longer, as planned by the patient’s body surface area).

### Site criteria (requirements for safety management system)

#### Safety management system

Given the peculiarities of this drug, a hospital or other medical facility conducting this clinical trial must satisfy the requirements related to the safety management system described in this section. This is to ensure that the therapy is provided by a team of medical personnel including physicians, radiology technologists managing radiation safety, and nurses providing patient care and assistance.

##### Establishment of safety management system

To ensure medical safety, safe handling of this drug, and radiation safety, the administrator of a hospitals or other medical facility conducting this clinical trial must make the radiation safety supervisor and the radiation safety officer involved in this clinical trial familiar with the contents of this manual. In addition, this clinical trial should be conducted under the following organizational medical safety management system of the hospitals or other medical facility.

##### Education and training related to radiation safety management

Prior to the conduct of this clinical trial, the radiation safety supervisor and the radiation safety officer (hereinafter referred to as “radiation safety supervisor, etc.”) involved in this clinical trial must complete the education and training shown in “4.3 Education and Training” of this manual to ensure the safe handling this drug and radiation safety.

##### Designation and responsibilities of a radiation safety supervisor

The administrator of a hospital or other facility conducting this clinical trial will designate a radiation safety supervisor for this clinical trial from physicians who have expertise to conduct this clinical trial and have completed the education and training. Usually, the sub-investigator of the clinical trial from nuclear medicine or radiology will assume the responsibility. The radiation safety supervisor will provide education on radiation safety management to health-care professionals involved in this clinical trial at the facility and will also supervise and oversee them from the viewpoint of radiation safety management of this clinical trial.

##### Designation and responsibilities of a radiation safety officer

The administrator of a hospital or other medical facility conducting this clinical trial will designate a radiation safety officer from radiology technologists, nurses, etc., who have expertise to conduct this clinical trial and have completed the education and training. The radiation safety officer will, under the supervision of the radiation safety supervisor, engage in radiation safety assurance and radiation safety management, etc., in this clinical trial.

### Site criteria (other matters to be complied with)

The following conditions must be satisfied to conduct this clinical trial according to this manual.

1. The clinical trial should be conducted based on a protocol that has been appropriately examined.
2. When an expert such as a radiation safety officer provides instructions such as precautions regarding this clinical trial to an eligible patient and his or her family members (or caregivers) prior to this therapy, the expert will determine whether the patient can function normally in line with these instructions. This therapy will be performed if the patient and his or her family members (or caregivers) can follow and consent to follow these instructions.
3. After discharge, a patient’s residence will be equipped with appropriate sewage and a flush toilet.
4. The patient is able to function normally in terms of decision-making and behavior.
5. Contact between the patient and infants and pregnant women will be minimized for 1 week after administration of this drug.

### Safety management of this product

#### Record keeping

When using ^90^Y, efforts should be made for safety management in accordance with the regulations stipulated in relevant laws and ordinances. ^90^Y must be handled and stored in an appropriate manner and the location must be clarified. For this reason, record keeping is specified for the following matters.

1. Records of the receipt, use, and disposal (Radiopharmaceutical Usage Records)

The following items are required on the usage records. (Article 30, Section 23, Subsection 2 of the Ordinance for Enforcement of the Medical Care Act, Notice No. 51, 1974, Medical Affairs Bureau, Ministry of Health and Welfare and HPB Notification No. 0315-4).

(1) Product specifications, (2) date of arrival, (3) date of use, (4) quantity used, (5) quantity remaining, (6) user, (7) name of patient, (8) date of storage or disposal, and (9) radioactivity during storage and disposal.

In addition, storage records will be kept and the quantity stored at the facility will be verified to ensure that the planned maximum storage quantity is not exceeded.

2. Measurement and recording in places where radiation injuries could occur

For places where radiation injuries may occur, the radioactivity and the status of contamination by radionuclides will be measured once prior to the start of this therapy and once in a period not exceeding 1 month (a period not exceeding 6 months for designated places) after the start of this therapy, and records of the results will be retained for 5 years.

The radioactivity will be measured in the area related to use (external side of the wall of room where medical radionuclides are used, room where RIs are used, storage room, disposal facilities, room for patients undergoing radiation therapy, boundaries of the controlled areas, living quarters, property lines of the hospital or clinic). The radioactivity will be measured using 1-cm dose equivalent (rate) (if the 70-μm dose equivalent [rate] may exceed ten times the 1-cm dose equivalent [rate], the 70-μm dose equivalent [rate] will be used).

The status of contamination by radionuclides will be measured for the room where RIs are used, room for patients undergoing radiation therapy, drainage ports of drainage facilities, exhaust ports of exhaust facilities, place of drainage monitoring facility, place of exhaust monitoring facility, and boundaries of controlled area.

The level of radiation and the status of contamination with radionuclides will be measured using a radiation-measuring device at the positions most suitable for measuring them. However, if it is significantly difficult to measure them with a radiation-measuring device, these values can be estimated by calculation (Article 30, Section 22 of the Ordinance for Enforcement of the Medical Care Act and Article 54 of the Ionizing Radiation Ordinance).

3. Calculation of exposure dose, effective dose and equivalent dose for radiology technologists and technicians

In accordance with Article 30, Section 18 of the Ordinance for Enforcement of the Medical Care Act, the dose from external exposure and internal exposure will be measured. Based on those measurements, the effective dose and equivalent dose for radiology technologists and technicians, etc., will be calculated as stipulated by the Minister of Health, Labour and Welfare (Notification No. 398 [15], from the Ministry of Health and Welfare dated December 26, 2000). (Article 8 of the Ionizing Radiation Ordinance)

4. Ionizing Radiation Medical Examination Personal Form

The results of the medical examination performed on workers who are always engaged in clinical radiology and enter the controlled area (radiology technologists and technicians) must be recorded in the “Ionizing Radiation Medical Examination Personal Form” and retained for 30 years (however, if the documents are delivered to an institution designated by the Minister of Health, Labour, and Welfare after 5 years of storage, the storage is exempted.) (Article 57 of the Ionizing Radiation Ordinance).

5. Records related to the release of a patient administered radiopharmaceuticals

When a patient is released or discharged in accordance with PMSB Notification No. 70, the following items will be recorded and retained for 2 years after the release.

1. Dose, date and time of release, dose rate measured at release
2. Precautions and guidance for mothers with nursing infants

#### Restrictions on places where radiopharmaceuticals can be used

A radiopharmaceutical must be handled in a room designed for radiopharmaceutical use (Article 30, Section 14 of the Ordinance for Enforcement of the Medical Care Act), and this drug should be used in the room where radiopharmaceuticals are used in principle. When using medical radionuclides (radiopharmaceuticals), it is necessary to establish storage facilities and disposal facilities (see section 2.2.1 Table 2).

The standards for these facilities are shown below in Article 30 Sections 8, 9, 11, and 12 of the Ordinance for Enforcement of the Medical Care Act and HPB Notification No. 0315-4, a related notification:

1. Standards for the room where medical radio nuclides are used:
  Article 30, Section 8 of the Ordinance for Enforcement of the Medical Care Act (room where medical radio nuclides are used)
2. Standard for storage facilities (storage box):
  Article 30, Section 9 of the Enforcement Regulations of the Medical Service Act (storage facilities)
3. Disposal facilities (standards for drainage facilities, exhaust facilities, and storage and disposal facilities):
  Article 30, Section 11 of the Enforcement Regulations of the Medical Service Act (Disposal facilities)
4. Standards for buildings and facilities of hospital rooms for patients receiving treatment with medical radionuclides:
  Article 30, Section 12 of the Enforcement Regulations of the Medical Care Act (Rooms for patients undergoing radiation therapy)

#### Control of exhaust, drain water and place of use, and concentration limit

For the use of radiopharmaceuticals, the radioactivity at the place of use and the status of contamination by radionuclides will be measured, and the quantity and concentration of radionuclides in the drain water or exhaust must be not exceed the concentration limit to ensure radiation safety (Article 30, Sections 22 and 26 of the Enforcement Regulations of the Medical Care Act and HPB Notification No. 0315-4, Section 6 [calculation of dose, etc.], 1) to 5).

For the use of ^90^Y as well, it is necessary to control the drain water and exhaust as well as to measure the concentration and dose rate, etc., of radioactive materials at the place of use as follows.

For locations related to the use of radiopharmaceuticals such as the rooms where medical radionuclides are used, surface contamination, 1-cm dose equivalent rate, and radioactive material concentration in air will be measured once in a period not exceeding 1 month, and records of the results must be retained for 5 years.

The sites that may be contaminated will be covered with water-absorptive polyethylene sheets, etc., in advance to prepare for possible contamination. In case of contamination, the contamination must be immediately removed, and whether the contamination has been surely removed must be confirmed by measurement.

Drainage should be performed after confirming by measurement that the sum of the ratio of the concentration of all radionuclides including ^90^Y in drainage water or drainage fluid to the concentration limit does not exceed 1.

Similarly, for the exhaust, confirmation should be made by measurement that the sum of the ratio of the concentration of all radionuclides including ^90^Y in the exhaust or air to the concentration limit does not exceed 1.

In principle, measurement of the radioactivity and the status of contamination by radionuclides will be performed using a radiation-measuring device. Estimation by calculation is limited to cases where it is physically difficult to measure them with a radiation-measuring device [14, 16].

Measurement of the exhaust and drain water can be entrusted to a reliable external company specialized in measurement only when the standards stipulated in Article 15, Section 2 of the Medical Care Act are complied with. The radiation safety supervisor, who clarifies the management system within each organization, is responsible for retaining records of obtained measurement results, etc., and grasping the control status even when the radiation measurement is outsourced. Also, the facility is responsible for managing the status.

## Preparation and administration of yttrium-90-labeled anti-P-cadherin antibody injection

### Composition and preparation of this drug

Prior to administration of this drug in the clinical trial, labeling of ^90^Y-anti-P-cadherin antibody injection should be performed at a hospital or other facility conducting the clinical trial. The investigational drug therefore consists of the following reagents and components.

- Anti-P-cadherin antibody solution vial.
- Buffer solution for preparation vial.
- Yttrium chloride solution (1850 MBq/mL, 1 mL).
- Sterile vial for preparation.

#### Labeling method of this drug (outline)

After appropriate measures for radiation protection and contamination prevention have been taken, labeling will be performed at the trial site in accordance with the “Labeling and preparation procedures (outline)” provided separately by the sponsor.

The labeling method is outlined below. If the dose (radioactivity) exceeds 1400 MBq, the following steps 2 to 4 will be performed twice.

1. Calculate the dose (radioactivity) of this drug based on the patient’s body surface area, scheduled dosing date, etc.
2. Add 1 mL of anti-P-cadherin antibody solution and an amount of yttrium chloride solution equivalent to 1480 MBq to a sterile vial for preparation set in a shielded container.
3. Place the shielded container of the mixture in the hot labeler set at 40 °C and heat at 40 °C for 990 s in a static condition.
4. After cooling, add the buffer solution for preparation to make 10 mL in total volume.

#### Confirmation of radiochemical purity

Confirm that the radiochemical purity is not less than 95% by the following method. Do not use if the radiochemical purity is less than 95%.

1. Spot 5 μL of the labeled solution on the starting line of a thin-layer plate (ITLC-SG strip) and develop the plate in a glass vial with 1 mL of the developing solvent (physiological saline).
2. Cut the ITLC strip at the cut line, place the upper part and the lower part, respectively, in the tubes for measurement, measure each radioactivity with a gamma counter, and determine the radiochemical purity.

#### Radioactivity administered

1. Measure the radioactivity of the sterile vial for preparation after labeling with a properly calibrated radiation-measuring device.
2. Collect the dose to be administered (radioactivity) in a syringe, and measure the radioactivity level of the sterile vial for preparation after the collection using a radiation-measuring device to calculate the radioactivity from the difference in the radioactivity before and after the collection. If necessary, adjust the volume of the solution in the syringe to get the radioactivity to be administered.

### Administration methods (outline)

This drug prepared with an appropriate radioactivity will be directly administered intravenously over at least 10 min, and the intravenous line will be flushed with physiological saline using a three-way stopcock. For administration, the exposure protection shown in “4.1.5 Protection from exposure during administration” should be performed.

## Protection from exposure

### Protection from exposure before administration (during preparation) and during administration

When preparing and administering this drug, the following precautions for radiation protection should be carefully considered and measures for radiation protection and prevention of contamination should be taken.

#### Basic matters

1. Wear working clothes and gloves and prepare and administer this drug with appropriate shielding equipment, with the knowledge that under normal glove thickness the beta rays released from ^90^Y is hardly shielded.
2. When handling radioisotopes (hereinafter referred to as RIs), it is necessary to make efforts to reduce exposure by minimizing the handling time of, maximizing the distance from, and shielding the beta rays.
3. Be familiarized with procedures by conducting cold runs repeatedly (practice of handling process without RI) and always perform a hot run (training actually using RI) before starting this therapy at a facility.
4. The sites that may be contaminated will be covered with water-absorptive polyethylene sheets, etc., in advance to prevent contamination and prepare for potential expansion of contamination.
5. In case of contact with skin such as hands and face, wipe immediately and wash thoroughly with running water. If the drug enters the eye, immediately wash the eye thoroughly with physiological saline or running water.
6. After the procedures, be sure to measure the surroundings with a beta-ray survey meter to confirm that there is no contamination.

#### Precautions for controlled areas

Precautions for entering and exiting controlled areas and laboratories will be posted near entrances and exits in accordance with the provisions of the Medical Care Act. Radiology technologists and technicians who work with radiation must be fully informed of these precautions. Key precautions are as follows:

1. An entry log will be kept.
2. Radiology technologists and technicians will change into footwears, athletic shoes, or protective shoes dedicated for use in the controlled areas.
3. Radiology technologists and technicians will change into dedicated work clothes for use in the controlled areas.
4. A personal dosimeter such as a dosimetry badge will be worn on the chest by males and on the waist by females.
5. The fact that ventilation equipment in a ventilation system is in operation will be verified.
6. When handling a radiopharmaceutical, protective eyewear and protective gloves must be worn.
7. A radiopharmaceutical or radioactive material that is discarded after use will be immediately transferred to a waste collection room once procedures are complete.
8. Radioactive contamination will be tested for indoors after use of a radiopharmaceutical or radioactive material. If contamination is found, decontamination will immediately be performed.
9. Hands will be washed and cleaned with cleanser and running water.
10. The hands, feet, shirt cuffs, surface of clothing, footwear, etc., will be tested for contamination.
11. If no contamination is present, personnel may change footwear and clothing. If contamination is found, decontamination will be performed in accordance with instructions from a radiation safety supervisor, etc.
12. An exit log will be kept.
13. Personal dosimeter readings will be recorded.

#### Preparation of protective equipment

1. Protective eyewear (required): Protective eyewear will be prepared since the injectable could directly contaminate the eyes when this drug is handled.
2. Wearing protective gloves (required): To avoid direct contamination of the fingers when handling this drug.
3. Water-absorbent polyethylene-coated filter paper: Polyethylene-coated filter paper to absorb moisture containing radioactive material to avoid the spread of contamination. Polyethylene-coated filter paper will be placed where contamination might occur, such as inside a biological safety cabinet, on surrounding work surfaces, and on lead bricks.
4. Forceps: Forceps with silicone-covered tips stop slippage and facilitate grasping of a vial with forceps.
5. A tray of an appropriate size: A stainless steel tray of an appropriate size will be covered with water-absorbent polyethylene-coated filter paper, and this drug will be dispensed over the tray. Even if radioactive liquid spills during handling, radioactive contamination will be limited to the tray, helping to prevent the spread of contamination.

#### Protection from exposure during preparation

1. Preparation before labeling and labeling operations will be performed in accordance with “Labeling preparation procedure.”
2. Conduct cold runs before the labeling operation.
3. Make sure to prevent accidents by organizing the labeling operation environment and being familiarized with the labeling preparation procedure. In addition, labeling must not be performed by one person alone.
4. Care should be taken to avoid inadvertent access of people to the place where labeling is performed and the place where high-concentration radioactive solution is handled.
5. Even during the operation, check for contamination from time to time with a survey meter as appropriate.
6. If contamination is found, take measures to prevent spread to the surrounding area and immediately decontaminate.

#### Protection from exposure during administration

1. The radioactivity to be administered should be determined in advance based on the patient’s body surface area and the radioactivity decay table
2. Vials and syringes should be handled with appropriate shielding devices. High energy beta rays released from ^90^Y produce bremsstrahlung radiation when hitting metals, so care must be taken to select the material of the shielding devices. For this reason, it is desirable to use acrylic, etc., of sufficient thickness to shield the beta rays or to use a shielding device combining acrylic, etc., on the inside and tungsten or lead, etc., on the outside of the device. When selecting metal shielding devices, make sure that they have sufficient thickness to block bremsstrahlung radiation.
3. When checking the radioactivity, promptly measure the vial taken out from the shielding device with a calibrated measuring instrument and immediately return it to the shielding device.
4. To prevent extravasation, this drug should be administered slowly (over at least 10 min) by direct intravenous injection after establishing a venous access with a butterfly needle, indwelling needle, etc., preferably by using a continuous infusion pump. Then flush the intravenous line with physiological saline from the same syringe using a three-way stopcock.
5. If the puncture into a blood vessel is wrongly performed at administration, this drug should be administered into a blood vessel in the opposite arm to prevent the leakage of radiation from the puncture site.
6. If extravasation is noticed, stop injection immediately, mark the leakage site, and promote diffusion by heating and massage.
7. After the procedures, measure the surroundings with a beta-ray survey meter, etc., and confirm that there is no contamination.

#### Protection from exposure during disposal of radioactive waste after administration

When handling radioactive waste after labeling and administration of this drug, protective equipment such as laboratory coat and gloves should be worn, and the radioactive waste should be immediately separated after completion of the operation to be stored until disposal.

### Testing for contamination and decontamination after administration

#### Testing for contamination and decontamination in rooms where this drug is used (walls and floor)

A radiation-measuring device will be used to test the inside of biological safety cabinets and the floor for contamination with this drug in accordance with the manner in which this drug is used.

Since ^90^Y emits beta rays only, after procedures, the surroundings should be measured with a beta-ray survey meter to confirm that there is no surface contamination. Preparation and dispensing other radiopharmaceuticals containing nuclides at the same time in a room where radiopharmaceuticals are used can lead to incorrect administration, and thus other radiopharmaceuticals should not be prepared and dispensed at the same time to ensure medical safety.

When measuring a contaminated site by ^90^Y, select an appropriate radiation-measuring device.

When radioactive contamination is found on a workbench or the floor, decontamination must be quickly performed. If contamination is found relatively early, the contaminant will be wiped up with paper towels and the site will be gradually decontaminated using water, a neutral detergent, or a chelating agent such as citric acid. These procedures are typical. Attention will be paid to tears or pin holes in the gloves that are used in decontamination to avoid secondary contamination of the body. If the site cannot be completely decontaminated, the extent of contamination, measurements, and the date of contamination will be clearly indicated with a permanent marker. Barriers preventing access to avoid the spread of contamination are an appropriate step to prevent exposure to radiation and to prevent contamination.

#### Exposure to medical personnel (external exposure and internal exposure)

Based on the Article 30 Section 18 and Article 30 Section 27 of the Ordinance for Enforcement of the Medical Care Act, Section 5 (Matters regarding limits) Subsections 1 and 2 and Section 6 (Calculation of doses) Subsections 1 to 5 of the HPB Notification No. 0315-4, medical facilities must strive to prevent the exposure of medical personnel (radiology technologists and technicians, etc.).

The dose of this drug depends on the patient’s body surface area. Table 3 shows the calculations of the dose from external exposure for radiology technologists and technicians based on the dose, time procedures take, and distance from the radiation source. Though the dose of this drug in Japanese clinical trials is assumed to be 925 MBq/m^2^ (up to 2220 MBq/dose)^1^ per patient’s body surface area, it was calculated conservatively under the condition that the labeling procedures with the dose of 1850 MBq will occur twice (3700 MBq). The effective dose rate constant used in dose evaluation is 0.00263 [μSv m^2^ MBq^−1^ h^−1^] as shown in Table 1. Safeguards must be taken to reduce the dose from external exposure in accordance with 4.1.

**Table 3:** Dose from external exposure to medical personnel

Stage	Effective dose (per procedure)			Skin dose (per procedure)			Dose limits	
	Time procedures take (min)	Distance (cm)	Exposure dose (mSv)	Time procedures take (min)	Distance (cm)	Exposure dose (mSv)	Effective dose limit (whole body)	Equivalent dose limit (skin)
Preparation	20	50	0.00218	20	1.0	5.46	Radiology technologists and technicians: 50 mSv/year 100 mSv/5 years Women of childbearing potential: 5 mSv/3 months	500 mSv/year
Administration	20	50	0.00218	20	1.0	5.46		

The effective dose (mSv/week) *E* of internal exposure of workers per week for personnel will be calculated using the formula below based on “Notification No. 398, [15] from the Ministry of Health and Welfare dated December 26, 2000.” (See the Manual on Management of Medical Radiation [17])

$$E = e \times I,$$

where *e* is the effective dose coefficient (mSv/Bq) and *I* is the quantity of a radiopharmaceutical (Bq) inhaled in 1 week.

$$I = 1.2 \times 10^{6} \times C \times t,$$

where 1.2 × 10^6^ is the air intake (cm^3^/h) of an adult in 1 h; *C* the average level of radioactivity in air (Bq/cm3) per week; *t* the time procedures take/week

$$C = A \times {\text{dispersal}}\,{\text{rate}} \times {\text{number}}\,{\text{of}}\,{\text{days}}\,{\text{of}}\,{\text{use}}\,{\text{in}}\,1\,{\text{week}}/(V \times \, 10^{6} \times \, 8\,({\text{h}}) \times {\text{number}}\,{\text{of}}\,{\text{days}}\,{\text{the}}\,{\text{ventilation}}\,{\text{system}}\,{\text{is}}\,{\text{in}}\,{\text{operation}}\,{\text{in}}\,1\,{\text{week}}),$$

where *A* is the planned maximum quantity (Bq) used in 1 day; *V* the indoor ventilation (m^3^/h) when the system in operation 8 h/day

When using this drug, *A* is 3700 MBq (maximum quantity used for administration of 2220 MBq), the dispersal rate is 0.001, the indoor ventilation in 1 day is 560 (m^3^/h) × 8 (h), the number of days of use in 1 week is 1 day (number of days of using this drug), the number of days of operation of the ventilation system in 1 week is 5 days, the time procedures take is 20 min (0.333 h), and *e* (effective dose coefficient when ^90^Y is inhaled) is 1.6 × 10^−6^ (mSv/Bq). The effective dose *E* (mSv) as a result of internal exposure per week will be as follows:

- *C* = 3700 × 10^6^ × 0.001 × 1/(560 × 10^6^ × 8 × 5) = 1.65 × 10^−4^ (Bq/cm^3^),
- *I* = 1.2 × 10^6^ × *C* × 0.333 × 1 = 65.93 (Bq),
- *E* = *e* × *I* = 1.6 × 10^−6^ × 65.93 = 1.05 × 10^−4^ (mSv) = 0.105 (μSv).

### Training

#### Training through the workshop on radiation safety handling

In this clinical trial, this therapy will be administered only to patients who are considered appropriate by a physician with adequate knowledge and experience in cancer treatment and radiotherapy. In addition, since a hospital or other facility providing this therapy in this clinical trial is required to acquire knowledge on the safety assurance of medical care and safety handling of radiation related to this therapy, the radiation safety supervisor, etc., designated for this clinical trial must have attended “Safety handling workshop for radioimmunotherapy using yttrium-90-labeled anti-CD20 antibody” held by the Japanese Society of Nuclear Medicine and related academic societies, from the viewpoint of radiation safety management related to internal radiotherapy using ^90^Y, the same nuclide of this therapy. Radiation safety management at the workshop for safety handling includes the following contents.

- Laws and ordinances, notification items, and release criteria.
- Safety management of radiopharmaceuticals for internal radiotherapy.
- Measurement of radioactivity and safe management of radioactive waste.

It is also desirable that physicians or pharmacists involved in preparation of this drug attend the workshop. The radiation safety supervisor will provide education and training to physicians or pharmacists involved in dispensing who have not attended the workshops for safety handling to make them understand this therapy and to deepen their knowledge about radiation safety management and actions for patients, etc.

#### Education and training on preparation of this drug

This therapy requires preparation of the drug at the trial site, and the drug with a radiochemical purity of 95% or higher should be administered. In addition to attending the workshop for safety handling on radiation safety management of internal radiotherapy using ^90^Y, prior to the preparation of this drug, the radiation safety supervisor, etc., and the physicians or pharmacists in charge of preparation must receive training on drug preparation based on the “Labeling preparation procedure” etc., provided by the sponsor.

#### Records of training

The radiation safety supervisor will prepare the implementation records of training conducted for this clinical trial. The implementation records will be retained for at least 2 years.

### Release of patients administered a radiopharmaceutical

The Article 30, Section 15 (admission restrictions), Subsection 1 of the Ordinance for Enforcement of the Medical Care Act stipulates that “the administrator of a hospital or clinic shall not admit a patient who is being treated^2^ with radiation-producing medical equipment or a radiation-producing medical device implanted or a patient who is being treated (see footnote 2) with a radiopharmaceutical or a positron emission tomography radiopharmaceutical to patient rooms other than rooms for radiation therapy.” Provisions of the Ordinance also seek to reduce the exposure of third parties, i.e., individuals other than the treated patient. That said, the same provisions “are not applicable when implementing appropriate safeguards and steps to prevent contamination.^3^” When a certain level of radiation protection is provided, the QOL of the treated patient will be taken into account and the administrator of a hospital or clinic is not necessarily obligated to admit a treated patient to a room for patients undergoing radiation therapy. This is the import of the guidelines for the “Release of Patients who have been Administered a Radiopharmaceutical.”

#### Release criteria for patients administered a radiopharmaceutical

The release criteria (PMSB Notification No. 70) have been laid out as guidelines to enhance the QOL of treated patients and to ensure the safety of the general public and caregivers from radiation. These criteria were publicized as an interpretation of the “proviso” stipulated in Article 30, Section 15, Subsection 1 of the Ordinance for Enforcement of the Medical Care Act. The gist of the release criteria is generally as follows:

1. Scope: When patients who have been administered a radiopharmaceutical are released or discharged from rooms where radiopharmaceuticals are used or rooms for patients undergoing radiation therapy in a hospital or other facility.
2. Release criteria: The “criteria for a reduced dose” stipulate that the dose for the general public will be 1 mSv each year.^4^Given the benefit for both patients and caregivers, the dose for caregivers will be 5 mSv^5^ per course of treatment.^6^
  In specific terms, a patient may be released or discharged when any of the following (1) to (3) apply:
  1. Release criteria based on dose
    A patient may be released or discharged when the dose or level of residual radioactivity in the body does not exceed the level of radioactivity as stipulated in the following table.
    **Table:** Level of radiation at the release or discharge of patients administered a radiopharmaceutical

Nuclides used in therapy	Dose or level of residual radioactivity in the body (MBq)
Strontium-89	200^a^
Iodine-131	500^b^
Yttrium-90	1184^a^

^a^Maximum dose

^b^The radioactivity of iodine-131 will be determined by combining the dose of external exposure from a patient’s body and the dose of internal exposure as a result of inhalation of iodine-131 expelled while the patient breathes
  2. Release criteria based on the measured dose rate
    A patient may be released or discharged when the dose rate measured at a point 1 m from the surface of the patient’s body does not exceed the value in the following table:
    **Table:** Dose rate for release or discharge of patients administered a radiopharmaceutical

Nuclides used in therapy	1-cm dose equivalent rate 1 m from the surface of the patient’s body (μSv/h)
Iodine-131	30^a^

^a^The dose equivalent rate will be determined by combining the dose of external exposure from a patient’s body and the dose of internal exposure as a result of inhalation of iodine-131 expelled while the patient breathes
  3. Release criteria based on calculation of the cumulative dose for per patient
    A patient may be released or discharged in the following situations based on the cumulative dose calculated per patient. (remainder omitted)
    **Table:** Examples where release criteria based on evaluation of the cumulative dose for each patient are satisfied

Nuclides used in therapy	Scope	Dose (MBq)
Iodine-131	Destruction of residual thyroid tissue (ablation) after a complete thyroidectomy to treat differentiated thyroid cancer with no distant metastasis^a^	1110^b^
Radium-223	Treatment of castration-resistant prostate cancer with bone metastasis^c^	12.1^d^ (72.6)^e^

^a^Conditions for treatment: It is limited to treatment performed in accordance with the treatment guide (“Outpatient Treatment with I-131 (1110 MBq) to destroy residual thyroid tissues”) created by relevant academic societies

^b^The radioactivity level of iodine-131 will be determined by combining the dose of external exposure from a patient’s body and the dose of internal exposure as a result of inhalation of iodine-131 expelled while the patient breathes

^c^Conditions for treatment: It is limited to treatment performed when radium chloride (R-223) injection is administered at 55 kBq/kg per dose at intervals of 4 weeks up to six times in accordance with the treatment guide (“Manual on the Proper Use of Internal Radiotherapy with Radium Chloride (Ra-223) Injection”) created by relevant academic societies

^d^Maximum dose per administration

^e^Maximum dose per treatment
3. Records of release
  When a patient is released, the following items will be recorded and the records will be retained for 2 years after the release.
  1. Dose, date and time of release, measured dose rate measured during release.
  2. Precautions and guidance for mothers with nursing infants
  3. When a patient is released based on (3) of 2) above, the method used to calculate the cumulative dose for that release (the rest omitted).
4. Precautions
  1. When a patient is released or discharged, the patient will be cautioned and instructed verbally and in writing with regard to his or her routine activities so as to avoid unnecessary exposure of third parties.
  2. If a patient is nursing an infant, the patient will be fully informed, cautioned, and instructed.
  3. Based on guidelines created by organizations such as academic societies and associations related to radiation, protection will be provided in accordance with the physical characteristics of the radionuclide, patients and caregivers, will be informed, and other steps will be taken to ensure safety from radiation and to safely manage radiation.

#### Factors related to the evaluation of release criteria

1. Exposure factor^7^: The duration of patient contact, the distance from the patient, and the radiation dose are elements of the dose from external exposure. Thus, the exposure factor is a factor that should be taken into account when evaluating the exposure dose to a third party. The exposure factor is determined by the extent of interaction with the patient.
  1. Exposure factor for caregivers: 0.5
    When a patient requires extensive care, the exposure factor is, based on measurements of the exposure dose for patients administered a radiopharmaceutical, reasonably set at 0.5 according to one study [19]. A Japanese study that measured the exposure dose from patients who had been administered radiopharmaceuticals also indicated that an exposure factor of 0.5 was appropriate [20]. Thus, an exposure factor of 0.5 is used to evaluate the dose for caregivers after a patient is released or discharged [21, 22].
  2. Exposure factor for the general public: 0.25
    In a typical home, an exposure factor of 0.25 is appropriate based on one study’s measurements of the exposure dose for a patient’s family members [19]. An exposure factor of 0.25 is used for family members other than caregivers and members of the general public after a patient is released or discharged [21, 22].

### Precautions after administration

#### Release of patients administered this drug

This manual explains the criteria for actually releasing patients treated with this drug from RI-controlled facilities, etc., in this study based on the “4.4 Release of patients administered a radiopharmaceutical.”

Regarding the application of “3. Release criteria (1) to (3)” of “Guidelines for Release of Patients Administered a Radiopharmaceutical” for ensuring radiation safety of third parties from patients treated with this drug, treatment with this drug at a dose of 925 MBq/m^2^/dose (maximum: 2220 MBq, 60 mCi) is planned to be given up to four times a year at intervals of 12 weeks or longer in the development in Japan of this drug. Since the radioactivity administered depends on the body surface area of the patient and the dose and frequency of administration may be reviewed according to the patient’s condition, it is considered appropriate to apply the “3. Release criteria (3) Release criteria based on calculation of the cumulative dose for each patient,” aimed at tailored treatment for the release criteria for patients administered this drug.

##### Exposure dose for a third party from a patient administered this drug

The exposure dose for third parties such as caregivers and the general public includes external exposure to radiation emitted from radioactive materials in the body of a patient administered this drug and internal exposure due to contamination from a patient’s excreta, etc. The dose to which a third party is exposed is comprehensively evaluated as follows:

##### Evaluation of the dose from external exposure

*Effective dose rate of external exposure at a distance of 1* *m from a patient administered this drug*

The formula for calculation of the dose rate for external exposure to a third party exposed to a patient administered this drug is

1 $$I = A \times C \times F_{a} \div L^{2} .$$

Here *I* is the effective dose rate [μSv/h] at a determined reference point; *A* is the residual radiation [MBq] in the body of a patient administered this drug; *C* is the effective dose rate constant for ^90^Y [μSv m^2^ MBq^−1^ h^−1^]; the value is 0.00263 [μSv m^2^ MBq^−1^ h^−1^] in 2.1.1 Table 1 will be used. *F*_*a*_ is the effective dose transmission rate (in case of multiple shielding, the overall product is taken as the transmission rate); *L* the distance [m] from the radiation source to the point of calculation.

*Cumulative dose to which a third party is exposed from a patient administered this drug*

The formula for calculation of the cumulative effective dose when a third party is continuously exposed to radiation from a patient administered this drug is

2 $$E = A \times \smallint_{0}^{\infty } \left( {\frac{1}{2}} \right)^{{\frac{t}{T}}} {\text{d}}t \times C \times f_{0} .$$

Here, *E* is the cumulative effective dose [μSv] to which a third party is exposed; *A* is the residual radiation [MBq] in the body of a patient administered this drug; *C* is the effective dose rate constant for ^90^Y [μSv m^2^ MBq^−1^ h^−1^]; the value is 0.00263 [μSv m^2^ MBq^−1^ h^−1^] in 2.1.1 Table 1. *T* is the physical half-life of ^90^Y; *f*_0_ is the exposure factor (caregivers, 0.5; the general public other than caregivers, 0.25)

*Factors for evaluation of the cumulative dose for caregivers and the general public from a patient administered this drug*

1. The cumulative dose to which a third party is exposed after a patient administered this drug is released or discharged will be calculated based on the effective dose rate at a distance of 1 m from the surface of the patient’s body.
2. Radiation in the body of a patient administered this drug depends on the effective half-life of ^90^Y, which involves both its physical half-life and in vivo dynamics of this drug. The biological half-life and effective half-life of this drug were calculated to be 87 h and 37 h, respectively, as a result of the administration of this drug at 925 MBq/m^2^ (*N* = 3) in the phase I clinical trial outside Japan. However, this result was derived from data from three patients with various cancers, and the biological half-life may be greatly affected by individual differences in humans and the degree of disease. Therefore, in this manual, the evaluation of cumulative dose to a third party after administration of this drug will be based only on the conservative physical half-life.
3. Based on the results of the phase I clinical trial outside Japan, the planned dose of this drug per patient’s body surface area is assumed to be 925 MBq/m^2^/dose (maximum: 2220 MBq, 60 mCi) administered up to four times a year at intervals of 12 weeks or longer in the Japanese clinical trial. The body surface area of a patient is calculated using the Du Bois equation [23]. The calculation result with the average height (167.2 cm) and body weight (65.8 kg) [24] in Japanese males aged 20 years or older in 2014 is 1.74 m^2^. In this case, the dose of this drug is 1610 MBq.

*Provisional calculation of cumulative dose of external exposure for caregivers and general public exposed to radiation from a patient administered this drug*

Estimation of cumulative dose of external exposure for caregivers and general public at a distance of 1 m from a patient administered this drug

1. Exposure of caregivers
  $$\begin{aligned} {\text{Cumulative}}\,{\text{dose}}\,{\text{of}}\,{\text{external}}\,{\text{exposure}} & = 2220\,[{\text{MBq}}/{\text{dose}}] \times 0.00263 \, [\upmu{\text{Sv}}\,{\text{m}}^{2} \,{\text{MBq}}^{ - 1} \,{\text{h}}^{ - 1} ] \\ & \quad \times 1.443 \times 24\, [ {\text{h}}/{\text{d]}} \times 2.67\,[{\text{d}}] \times 0.5 \times 4\,[{\text{dose}}/{\text{treatment}}] \\ & = 1.080\,[{\text{mSv}}/{\text{treatment}}]. \\ \end{aligned}$$
  Here, 2220 [MBq/dose] is the maximum dose of this drug for one time per patient; 0.5 is the exposure factor for caregivers; 0.00263 [μSv m^2^ MBq^−1^ h^−1^] is the effective dose rate constant of ^90^Y; 2.67 [d] is the physical half-life of ^90^Y; 4 [dose/treatment] is the maximum number of doses administered to treated patients in the clinical trial.
2. Exposure of the general public
  $$\begin{aligned} {\text{Cumulative}}\,{\text{dose}}\,{\text{of}}\,{\text{external}}\,{\text{exposure}} & = 2220\,[{\text{MBq}}/{\text{dose}}] \times 0.00263 \, [\upmu{\text{Sv}}\,{\text{m}}^{2} \,{\text{MBq}}^{ - 1} \,{\text{h}}^{ - 1} ] \\ & \quad \times 1.443 \times 24\, [ {\text{h}}/{\text{d]}} \times 2.67\,[{\text{d}}] \times 0.25 \times 4\,[{\text{dose}}/{\text{treatment}}] \\ & = 0.540\,[{\text{mSv}}/{\text{treatment}}]. \\ \end{aligned}$$
  Here, 0.25 is the exposure factor for the general public.

##### Evaluation of the dose from internal exposure

Excreta from a patient administered this drug flows into a sewage treatment plant, which then flows into a river. It is estimated that ^90^Y excreted from a patient is separated from this drug and exists in the form of insoluble compound taking into account the chemical properties of yttrium. However, the possibility cannot be ruled out that ^90^Y remains in the form of soluble chelate compound without separation from this product and ^90^Y is taken as drinking water after reprocessing. Therefore, for provisional calculation of the dose from the internal exposure due to oral intake by a third party, it was assumed that all of the radiation administered to a patient flows into a river and ^90^Y would be present in a water-soluble form. The Yodo River water system, which accepts a large quantity of treated effluent, was used as an evaluation model.

- The average flow of the Yodo River water system: about 4.1 [TL/year].
- Population of the metropolitan Osaka area that obtains potable water from the water system: about 13.935 million (2015) (Osaka Prefecture + Nara Prefecture + Wakayama Prefecture + 1/2 of Hyogo Prefecture) [25].
- Total population of Japan: about 127.095 million (2015) [25].
- Population of the metropolitan Osaka area as a proportion of Japan’s total population: 10.96% (0.11).
- Number of patients with P-cadherin-positive recurrent solid cancer (ovarian cancer, bile duct cancer, head and neck cancer) in Japan: about 45,000 [person/year].
- Of the above, patients administered this product are estimated to be 10% or less: 4500 [person/year].
- Number of patients to be treated in the metropolitan Osaka area: 4500 × 0.11 = 495 patients (calculated by population ratio).
- Of note, 0.11 is the population ratio of the metropolitan Osaka area. Furthermore, it is assumed that one patient will receive 2220 MBq of this drug four times a year.
- Level of radioactivity of the total dose of this drug administered to patients in Osaka:
  2220 [MBq/dose] × 4 [time/person] × 495 [person] = 4.396 [TBq].
- It is assumed that all of this drug is discharged into the Yodo River water system, and that all of them exist in a water-soluble form.
- Concentration of this drug in the river:
  4.396 [TBq/year] ÷ 4.1 [TL/year] = 1.072 [Bq/L].
  Here, 4.1 TL is the annual average flow of the Yodogawa River water system.
- Annual intake of this drug per member of the general public (assuming 2 L of water for drinking per day) [26]:
  $$1.072\,[{\text{Bq}}/{\text{L}}] \times 2\,[{\text{L}}/{\text{day}}] \times 365\,[{\text{day}}/{\text{year}}] = 782.6\,[{\text{Bq}}/{\text{year}}].$$
- In the aforementioned instance, the dose from internal exposure in 1 y:
  782.6 [Bq/year] × 2.7 × 10^−6^ [mSv/Bq] ≈ 2.11 [μSv/year].
  Here, 2.7 × 10^−6^ [mSv/Bq] is the effective dose coefficient [15] for oral intake of ^90^Y.
- The internal exposure dose of 2.11 μSv per year is 0.21% of 1 mSv per year, the ICRP recommended exposure dose limit for the general public.

##### Comprehensive evaluation of the doses from external and internal exposure

- Exposure dose for caregivers = 1.080 [mSv] + 2.11 [μSv] = 1.082 [mSv].
- Exposure dose for the general public = 0.540 [mSv] + 2.11 [μSv] = 0.542 [mSv].

Thus, the cumulative dose to which caregivers and the general public are exposed from a patient who received 2220 MBq of this drug up to four times for this therapy was lower than the dose to be restricted for caregivers and the general public (caregivers: 5 mSv/case, general public: 1 mSv/year). In this case, the release criteria for a patient administered this drug are considered to be satisfied. Therefore, since this patient meets the safety guidelines for release, the patient can be released and discharged from the RI-controlled facility, etc., immediately after administration of this drug.

However, when release and discharge are allowed, it is mandatory to provide precautions and instructions in writing and orally to ensure radiation safety in daily life, etc. Therefore, it is necessary to retain records showing that such explanation was given to the family members and the patient and their consent was obtained.

##### Criteria for release of a patient administered this drug from RI-controlled facility, etc.

The criteria for release of a patient administered this drug in the clinical trial of this drug in P-cadherin-positive recurrent solid cancer are as follows:

1. Nuclide used for treatment: ^90^Y.
2. Dose: 2220 MBq
  Maximum dose per administration. However, this therapy will be administered up to four times at a dose of 925 MBq/dose per patient’s body surface area (m^2^).

##### Example of assessment of external exposure to family members (caregivers) and the general public

External exposure dose from a patient in various daily situations is calculated under the following conditions and is shown in Table 4. The dose of this drug (*A*) was set at 2220 MBq (maximum dose per administration), the effective dose rate constant (*C*) at 0.00263 μSv m^2^ MBq^−1^ h^−1^ (effective dose rate constant of bremsstrahlung radiation against a target of atom number 20 by beta rays from ^90^Y) [27], and the physical half-life at 2.67 days.

**Table 4:** Calculation example of external exposure dose from patients in various daily situations

	Distance (m)	Time (h/day)	Frequency (time/week)	Exposure dose (mSv)	
				(1 dose)	(4 doses/year)
In-home contact	1	6	7	0.136	0.546
Sleeping in the same room	1	8	7	0.182	0.728
Third party in the workplace	1	8	5	0.130	0.520
Third party when commuting	0.3	1	5	0.181	0.722

As shown in Table 4, the exposure dose for family members (caregivers) (exposure factor 0.25) is 0.136 mSv/dose (0.546 mSv for 4 doses at a maximum) when exposed to the patient for 6 h every day at a distance of 1 m, and 0.182 mSv/dose (0.728 mSv for 4 doses at a maximum) when sleeping for 8 h every day. Provisional calculation based on physical half-life also estimates that in normal contact, the exposure dose will not exceed 1 mSv/year, acceptable dose for the general public.

### Precautions for patients and their family members (caregivers)

It is necessary to provide clinical information and pay attention to patients who have received this therapy and their family members, as well as to prevent unnecessary exposure of the patient’s family members (caregivers) and the general public due to this therapy during the release of the patient. As for the effect of the administered radiation on the surroundings, the external exposure dose of the family members (caregivers) is not more than the standard dose to be restricted even if the patient is physically contacted or care is provided close to the patient.

However, instruction should be given to avoid prolonged or near contact for a certain period after administration. This is because the release criteria based on the dose and dose rate specified in the PMSB Notification No. 70 are based on the conditions of contact with patients. For example, as a condition of contact with a patient, the exposure of the general public is specified as 25% of the annual exposure received by a third party at a distance of 1 m from the patient (it is equivalent to the contact with the patient for 6 h a day at a distance of 1 m). Instructions should be given to avoid contact in time exceeding this or nearer distance.

Therefore, the following precautions should be explained to patients and their family members (caregivers) in writing prior to administration to obtain their understanding of measures for radiation exposure reduction and contamination protection for third parties.

#### Precautions for 3 days after administration of this drug

Because relatively high radioactivity is present in blood, urine, etc., for a certain period after administration, patients should be instructed to pay attention to the following points especially for 3 days after administration and to ensure their thorough implementation.

[Precautions for daily activities]

1. If a patient loses blood, that blood will be wiped up with toilet paper and flushed down the toilet.
2. When there is any potential for coming into contact with a patient’s urine or feces and when coming into contact with clothing contaminated by a patient’s urine or feces, disposable latex gloves will be worn.
3. When a patient’s bodily fluids such as blood come into contact with the hands or skin, the contaminated site will be immediately washed with soap.
4. Sexual intercourse should be refrained from. Also, contraception should be used for 12 months after administration.
5. Prolonged or near contact with family members, spouse, child, or public should be avoided as much as possible (in particular, contact with children and pregnant women should be minimized).
6. Shower every day as much as possible. Regarding bathing, take a bath last and alone, and wash the bathtub immediately after bathing.
7. Take sufficient water.

[Precautions with regard to handling laundry]

1. Clothing worn by a patient administered this drug will be washed separately from the clothing of other individuals, and not at the same time. In addition, bed linens and undergarments soiled with blood or urine will be pre-washed well.

[Precautions with regard to urination, defecation, or vomiting]

1. Male patients will urinate while seated.
2. When feces or urine soil the toilet or floor, the material will be wiped up with toilet paper and flushed down the toilet.
3. A toilet will be flushed about two times after use.
4. Hands will be washed and cleaned with soap after urination or defecation.
5. Hands and skin that come into contact with a patient’s bodily fluids (e.g., blood), excreta, or vomitus will be cleaned and washed with soap.

#### Radiation safety management for a patient wearing a diaper or using a urinary catheter

The following precautions must be taken by a patient who is wearing a diaper or using a urinary catheter soon after administration (in principle, 1 week).

When handling a diaper, urinary catheter, or urine collection bag, disposable gloves will be worn, i.e., the same precautions as are followed to prevent biohazards will be taken.

[Precautions when wearing a diaper or using a urinary catheter (at home or in the hospital):]

1. Vinyl sheets should be used by an incontinent patient who is wearing a diaper.
2. When a patient is still using a urinary catheter despite being released from a room for patients undergoing radiation therapy, the urine in a urine collection bag will be disposed of in the toilet and the toilet will be flushed twice. After handling, hands will be washed and cleaned.
3. A catheter or urine collection bag will be replaced prior to the discharge of an inpatient.

[Precautions during disposal of a diaper or urinary catheter]

1. A diaper worn by a patient at home will be placed in a plastic bag and the bag will be closed so that the contents do not leak. The bag will be disposed of as ordinary garbage.
2. When disposing of infectious waste such as diapers at a hospital, refer to Handling the Diapers of Patients who have been Administered a Radiopharmaceutical (Guidelines for Medical Personnel Working in Nuclear Medicine) (March 2001, 1st ed., March 2004, 2nd ed.) [28].

### Precautions for medical personnel

Medical personnel involved in this therapy should fully understand this manual and the in vivo dynamics of this drug, and explain the above-mentioned principles for radiation protection to patients and their family members in an easy-to-understand manner. In addition, physicians with expertise about this clinical trial should provide appropriate education and training to medical personnel to improve the cooperation system at the medical institution. If urgent medical treatment is required, appropriate medical treatment may be prioritized over the above compliance with radiation protection to save the life of patient, etc.

Because relatively high radioactivity may be present in blood, urine, etc., for a certain period after administration, attention should be paid to the following points for 3 days after administration, particularly for those who are engaged in nursing care of patients.

1. Wear disposable gloves if there is a possibility of contact with the patient’s urine, feces, or blood and when handling clothes contaminated with them.
2. Wash hands thoroughly after touching the patient’s excreta, blood, etc., or after work.
3. Wash clothes contaminated with patient’s excreta, blood, etc., separately from clothes of other people.

## Disposal of radioactive contaminants from medical sources

Objects contaminated with ^90^Y fall under “radioactive contaminants from medical sources” specified in Article 30, Section 11 of the Ordinance for Enforcement of the Medical Care Act. Radioactive contaminants from medical sources will be stored and disposed of at the disposal facilities of each institution in accordance with the provisions of Article 30, Section 11 of the Ordinance for Enforcement, and may be disposed of by a party designated by the ordinance of the Ministry of Health, Labour and Welfare in accordance with the provisions of Article 30 Section 14, Subsection 2-1 of the Ordinance for Enforcement. Currently, the Japan Radioisotope Association is the only party to whom disposal is entrusted. The Japan Radioisotope Association collects radioactive contaminants according to the Entrustment Rules for RI Waste Disposal [29] as the sole trustee of disposal of radioactive contaminants from medical sources from each institution.

Waste materials generated in association with the use of ^90^Y are to be separated from RI wastes contaminated with other nuclides. Therefore, they are stored in dedicated ^90^Y waste storage containers (blue). It is not necessary to separate combustible materials, fire-retardant materials, and non-combustible materials by type. The residual solution in vials of this drug can be stored in the blue ^90^Y waste storage containers as it is without being drained to the RI drainage facility. The container will be placed in a 50 L drum (green, flame retardant) and will be stored and disposed of in the storage and disposal facility. For details, see the pamphlet of Japan Radioisotope Association “Collection of RI waste” and “Separate storage of medical RI waste contaminated with ^90^Y”.

Attention should be paid to the fact that the Japan Radioisotope Association cannot collect items with excreta from the human body or blood attached such as diaper and urine bag. A diaper or urine collection bag soiled with human excreta or blood will be handled in accordance with “Handling the Diapers of Patients who have been Administered a Radiopharmaceutical (Guidelines for Medical Personnel Working in Nuclear Medicine)” and “Manual on Handling the Diapers of Patients who have been Administered a Radiopharmaceutical (Japanese Society of Nuclear Medicine, Japan Radiological Society, Japanese Society of Radiological Technology, Japanese Society of Nuclear Medicine Technology, and Japan Association on Radiological Protection in Medicine) [25].

## Footnotes

1 Estimated dose based on the results of a phase I clinical trial conducted in the US.

2 “being treated” refers to patients who are treated with a medical irradiation equipment or device inserted into the body or treated with a medical radionuclide or a radionuclide for positron emission tomography for medical use and radiation exposure of patients other than the patient receiving radiation therapy may exceed 1.3 mSv per 3 months, according to Section 3 Subsection 11 (1) of HPB Notification No. 0315-4.

3 The proviso “Appropriate protective measures and contamination control measures” is specifically described in (A) through (C) of Section 4 Subsection 3 (3) of the notification.

(A) When a patient is released from the room for patients undergoing radiation therapy to the general hospital room, etc., the effective dose to other patients must be 1.3 mSv or less per 3 months.
  For handling of release of patients administered a medical radionuclide, see “Release of Patients who have been Administered a Radiopharmaceutical” (Notification No. 70 by the Director of the Safety Division, Pharmaceutical and Medical Safety Bureau, Ministry of Health, Labour and Welfare dated June 30, 1998, hereinafter referred to as “Pharmaceutical Release Criteria”). For handling of release of patients with permanent insertion of a medical irradiation device, see "Release of patients with permanent insertion of a medical irradiation device and handling of the radiation source after the insertion" (Notification No. 0710-1 by the Director of the Medical Care Planning Division, Health Policy Bureau, Ministry of Health, Labour and Welfare dated July 10, 2018, hereinafter referred to as "Irradiation Device Release Criteria”). It should be ensured that instructions to patients and caregivers, etc. are provided and that the release is recorded.
  Regarding the handling of release of patients who have administered a medical radionuclide that falls under Section 8 (c) and (d) of the Article 24, which apply to Article 24, Section 8 Subsection 2 of the Ordinance for Enforcement, see the Pharmaceutical Release Criteria and documents related to the calculation of release criteria in the “Release of Patients who have been Administered a Radiopharmaceutical” (Office communication of the Safety Division, Pharmaceutical and Medical Safety Bureau, Ministry of Health and Welfare dated June 30, 1998).
(B) (Omitted)
(C) (Omitted)

4 Dose limit for the general public: 1 mSv/year ICRP Publication 60 (1990 recommendation)^4)^ is used for dose limit for the general public (effective dose rate of 1 mSv per year. However, in special circumstances, a higher value may be acceptable in a single year under the condition that the average for a given 5-year period does not exceed 1 mSv per year). Currently, although it is not included in Japanese laws and regulations, the value described in ICRP Publication 103 (2007) [18], a new recommendation, has not been changed.

5 Cumulative dose for caregivers: 5 mSv Regarding the exposure dose to caregivers, volunteers, etc., in Section 95 of ICRP Publication 73 (1996) “Radiation Protection and Safety in Medicine”^5)^, the exposure of friends and relatives who assist in patient care and consolation is positioned as the medical exposure, and the following description is added: “The dose constraint of about several mSv per case is reasonable.” On the other hand, International Basic Safety Standards (1996)^6)^ of the International Atomic Energy Agency (IAEA) recommend practical values for dose constraint and dose limit for the patient’s comforter and visitor and also recommend that “The dose limits set in this part do not apply to patient’s comforter, i.e., individuals or visitors to the patient who are knowingly exposed while voluntarily (not employed or occupational) helping with nursing, attending, and comforting patients. However, the dose of any comforter or visitor should be constrained so as not to exceed 5 mSv per activity during patient’s diagnosis or treatment. The dose of a child visiting a patient taking a radioactive material must be similarly restricted below 1 mSv.”.

6 In an Office Communication (Data related to calculation of release criteria: Safety Division, Pharmaceutical and Medical Safety Bureau, Ministry of Health, Labour and Welfare, dated June 30, 1998) [19] issued at the same time as PMSB Notification No. 70, the cumulative γ-ray doses (the change of the radioactive material in the body released from the treated patient is the cumulative dose at a distance of 1 m from the source when only the physical half-life of the nuclide is taken into consideration) for 8 radiopharmaceutical nuclides commonly used in Japan at that time exceeded 20 mSv for I-131 (administered dose 1,110 MBq, exposure factor = 1) and 0.02–0.28 mSv (exposure factor = 1) for other radiopharmaceutical nuclides for diagnosis. Hence, release criteria were established for patients receiving therapeutic radiopharmaceuticals.

7 Exposure factor: Ratio of cumulative dose estimated to be actually received by a third party other than the patient in consideration of the time and distance in contact with the patient to cumulative dose when the patient stays at a distance of 1 m from the point source of the nuclide (in this case, the patient) for infinite time (time to decay of all nuclides).
